# The ACTTION Guide to Clinical Trials of Pain Treatments, part II: mitigating bias, maximizing value

**DOI:** 10.1097/PR9.0000000000000886

**Published:** 2021-01-21

**Authors:** Robert H. Dworkin, Robert D. Kerns, Michael P. McDermott, Dennis C. Turk, Christin Veasley

**Affiliations:** a Departments of Anesthesiology and Perioperative Medicine, Neurology, and Psychiatry, Center for Health + Technology, University of Rochester School of Medicine and Dentistry, Rochester, NY, USA; b Departments of Psychiatry, Neurology, and Psychology, Yale University, New Haven, CT, USA; c Departments of Biostatistics and Computational Biology and Neurology, Center for Health + Technology, University of Rochester School of Medicine and Dentistry, Rochester, NY, USA; d Department of Anesthesiology and Pain Medicine, University of Washington, Seattle, WA, USA; e Chronic Pain Research Alliance, Milwaukee, WI, USA

## Abstract

Summaries of the articles included in part II of the ACTTION Guide to Clinical Trials of Pain Treatments are followed by brief overviews of methodologic considerations involving precision pain medicine, pragmatic clinical trials, real world evidence, and patient engagement in clinical trials.

“…strategies that improve the efficiency of randomized trials and still protect their validity await rigorous investigation. Such investigations ought to be a high priority for clinicians and methodologists alike.”

—David L. Sackett, 1980^49^

## 1. Introduction

In this article, we introduce part II of the ACTTION Guide to Clinical Trials of Pain Treatments and discuss several important issues that are not covered elsewhere in the Guide. As we noted in the introduction to part I, the ACTTION Guide consists of a series of articles that is intended to serve as a basis for designing, conducting, analyzing, interpreting, and reporting the results of randomized clinical trials (RCTs) of treatments for acute and chronic pain.

In this section, we briefly summarize the 5 articles that are included in part II of the Guide, which begins with a comprehensive review and discussion of critical aspects of the design and conduct of confirmatory clinical trials of treatments for chronic pain. Katz^29^ brings an important perspective to this topic, having developed and implemented various methods for improving the quality of analgesic clinical trials. Confirmatory RCTs—often referred to as phase 3 trials—are typically conducted after preliminary evidence of a treatment's efficacy and safety has been found in early phase studies. Such clinical trials play a pivotal role in evaluating whether the evidence of a treatment's efficacy and safety provides an adequate basis for its use in clinical practice. Although a great deal of attention has been devoted to addressing sources of bias in RCTs, there is little doubt that continuing efforts to improve the quality and assay sensitivity of confirmatory trials of pain treatments have the potential to accelerate the development of pain treatments with greater efficacy and safety.

Few investigators, clinicians, or patients are completely satisfied with existing methods for assessing pain intensity. Despite the limitations of numerical and visual analogue scales, they have an impressive track record as primary outcome measures in RCTs of treatments for both acute and chronic pain. Patel et al.^42^ discuss ratings of pain intensity but also review the other clinical outcome domains and measures that complement pain intensity and, taken together, can provide a more comprehensive assessment of the patient's experience of chronic pain and of the efficacy and effectiveness of pain treatments. In virtually all circumstances, clinical trials of chronic pain should examine the effect of treatment on physical and emotional function, sleep, and patient global assessments of improvement or satisfaction, as well as other outcomes depending on the specific intervention and pain condition, as has been emphasized for almost 20 years.^61^

The ultimate goal of every clinical trial is to answer a question about a treatment or the condition being treated, most often by collecting data about efficacy or safety, but also by examining other features of the treatment and its implementation, for example, feasibility, patient adherence, or cost-effectiveness. Once such data are obtained, they undergo statistical analysis and interpretation. Dworkin et al.^10^ provide an overview of essential statistical principles of clinical trials for nonstatisticians who collaborate with biostatisticians in designing, conducting, and interpreting clinical trials of pain treatments and also for clinicians seeking to better understand the data analyses in published RCTs and their implications for clinical practice. Knowledge of the statistical aspects of research design, endpoints, sample size determination, missing data and trial estimands, data monitoring and interim analyses, and interpretation of results provides a valuable foundation for designing and evaluating clinical trials.

Most pain clinical trials have examined treatments that are hypothesized to reduce pain and/or improve function. Unfortunately, there has been relatively limited attention to studying interventions that have the potential to prevent the transition from acute pain to chronic pain. Interest in identifying the mechanisms of this transition^47^ and in preventing it from occurring has emerged over the past decade. Because prevention has not been sufficiently addressed elsewhere in the ACTTION Guide, we have reprinted an article that discusses key considerations for the design of clinical trials of interventions intended to prevent the development of chronic pain. Gewandter et al.^23^ discuss the prevention of 4 different pain conditions: chronic postsurgical pain, postherpetic neuralgia, chronic low back pain, and painful chemotherapy-induced peripheral neuropathy. Although some of what the authors discuss is specific to these conditions, many of the considerations can be extrapolated to RCTs designed to test preventive interventions for other chronic pain conditions.

There are few if any issues involving clinical trials of chronic pain treatments that are of greater current interest than the identification of subgroups of patients that are hypothesized to respond more robustly to a given treatment than other patient subgroups. Most of the research seeking to identify the characteristics of such patient subgroups has relied on patterns of symptoms and signs, assessed using self-report measures and quantitative sensory testing. Because the methods for assessing such phenotypes and examining their role in treatment response are not a focus elsewhere in the ACTTION Guide, we have reprinted an article that discusses the most promising phenotypic characteristics to examine in chronic pain RCTs, including psychosocial factors, symptom characteristics, sleep patterns, responses to noxious stimulation, endogenous pain-modulatory processes, and response to pharmacologic challenge. In this article, Edwards et al.^12^ provide evidence-based recommendations for core phenotyping domains and recommend measures of each domain. Although not reprinted in the ACTTION Guide, Smith et al.^55^ in a related article review the research on 3 important pain biomarkers—sensory testing, skin punch biopsy, and brain imaging—and discuss the different roles that each of these can play in clinical trials of pain treatments.

## 2. Trials, transitions, and translations

One of the major components of ACTTION is the Initiative on Methods, Measurement, and Pain Assessment in Clinical Trials (IMMPACT), the mission of which is to develop consensus reviews and recommendations for improving the design, execution, and interpretation of clinical trials of treatments for pain. The 2 articles on prevention and phenotyping that appear at the end of this section of the ACTTION Guide are based on IMMPACT meetings, as are 2 other recent articles on the conduct of analgesic trials and data quality^22^ and the interpretation of chronic pain clinical trial outcomes.^56^ There are other IMMPACT articles in preparation that present recommendations for clinical trials of opioid sparing, spinal cord stimulation, and visceral and pelvic pain, as well as for benefit-risk evaluation and reporting in chronic pain trials, which when published will provide important complementary information to the articles in the ACTTION Guide. In the following sections, we briefly discuss 3 other forthcoming IMMPACT efforts that reflect important developments in clinical research that are occurring across many different therapeutic areas and that are of great relevance for pain treatments.

### 2.1. Precision pain treatment

There has been markedly increasing attention devoted to developing personalized or precision treatments for a wide range of medical conditions. Precision treatment involves classifying individuals into subpopulations that differ in their responses to a specific treatment.^40^ We use the term precision treatment because it has been suggested that it is preferable to personalized medicine, which is sometimes misinterpreted as implying that unique treatments can be designed for each individual.^40^

The objectives of precision treatment are especially relevant to pain because there are numerous acute and chronic pain conditions with diverse and only partially overlapping pathophysiologic and psychosocial mechanisms. A major focus of ongoing research efforts is developing approaches to identify which patients respond best to a given pain treatment and then testing these predictions prospectively in RCTs. Such precision pain treatments would be based on phenotypes and biomarkers of the types discussed by Edwards et al.^12^ and Smith et al.,^55^ as well as any genetic factors that contribute to differences among patients in the mechanisms of their pain and in their responses to treatment.

The development of efficacious precision pain treatments involves challenging methodologic and statistical issues, foremost among which is avoiding the widespread assumption that variability in patient responses to treatment in a standard parallel group or cross-over trial is, in and of itself, evidence of true heterogeneity of the treatment effect. These trial designs, however, do not make it possible to separate random variation from true heterogeneity in patient responses to treatment; this requires other approaches, including multiperiod cross-over designs in which patients receive each treatment on at least 2 occasions.^11,25,51,52^ Other important challenges for the development of precision pain treatments include the need to design confirmatory trials in which hypotheses involving specific associations between the magnitude of the treatment effect and genotypes, phenotypes, or biomarkers are prespecified and tested with adequate control of the type I error probability,^9^ and the need to ensure a large enough sample size so that there is adequate statistical power to detect these associations (treatment-by-covariate interactions).^26^ Although the outlook for developing precision pain treatments is promising, considerable and sustained efforts will be required for success.

### 2.2. Translation to clinical practice

Over 50 years ago, Schwartz and Lellouch^50^ distinguished explanatory from pragmatic clinical trials, a similar but not identical distinction to that between efficacy and effectiveness trials. One major aspect of both of these distinctions is the contrast between the internal validity of the trial and its assay sensitivity to show the efficacy of a truly efficacious treatment vs the external validity of the trial to provide results that can be generalized to the effectiveness of the treatment in clinical practice. These 2 objectives of RCTs may be in competition,^49^ and as anyone who has been involved with designing or conducting clinical trials recognizes, the demographic and clinical characteristics of the patients enrolled may not reflect those who are seen in clinical practice. There is ample evidence of this across multiple therapeutic areas,^30^ and even a cursory examination of eligibility criteria confirms that this is also true of phase 2 and 3 RCTs of pain treatments.

Pragmatic trials are designed to maximize generalizability and (1) include patients who are similar to those who would receive the treatment if it were available in clinical practice and who are drawn from usual care settings, (2) allow greater flexibility in treatment delivery and adherence, and (3) prioritize outcome measures that are directly relevant to patients and not biomarkers or surrogate endpoints.^18^ Such trials often address questions involving the comparative effectiveness of different treatments,^15^ for example, comparing the effectiveness of 2 medications with different mechanisms of action or comparing the combination of a medication and a nonpharmacologic treatment with the medication alone. Some but not all pragmatic and comparative effectiveness trials include a usual care or other control group, which can be especially important when the assay sensitivity of the trial to detect group differences cannot be assumed.

Pragmatic trials are typically designed to maximize the external validity and generalizability of their results,^21,36^ and it can be challenging or even impossible for such trials to implement randomization, allocation concealment, and blinding of study staff and participants. Pragmatic trials can therefore be more subject to bias than efficacy trials that prioritize internal validity, and attention must be paid to addressing a variety of methodologic, statistical, and regulatory issues in their design and analysis.^27,38,59^ Although the value of high-quality data that can be generalized to clinical practice cannot be disputed, there has been limited consideration of the design and execution of pragmatic and comparative effectiveness trials of pain treatments.^48^ Fortunately, ongoing initiatives that involve collaborations among diverse stakeholders have the potential to substantially increase knowledge of the effectiveness of pain treatments in the community.^31^

Pragmatic clinical trials often make use of real-world data drawn from routine clinical practice, including information contained within electronic health records.^3,63^ However, such real-world data are increasingly being used in studies that are not clinical trials but that are intended to provide real-world evidence of treatment effectiveness and safety. When there has been no randomization to different treatments, the process of drawing causal inferences about a treatment becomes especially difficult because of the potential effects of known and unknown confounders. Differentiating “useful from misleading evidence in observational research”^28^ and determining “when and how can real world data analyses substitute for randomized controlled trials”^20^ is not only of great concern to investigators, patients, and clinicians but also to regulatory agencies.^8,45,54^ There are many crucial questions about pain treatments that real-word data have the potential to address—perhaps especially evaluating long-term safety and effectiveness—and it seems likely that the application of principled statistical methods^1,44^ to real-world data will lead to important advances in the treatment of both acute and chronic pain.

### 2.3. Patient engagement

The importance of involving patients in every aspect of clinical research is now universally recognized. Patient engagement in clinical trials includes participation in identifying unmet research needs; selecting and prioritizing outcome measures; collaborating on study design, execution, analysis, and interpretation; and translating, disseminating, and implementing clinical trial results in the community.^43,53^ Such patient engagement has been recommended and implemented across multiple therapeutic areas, including cardiology,^13^ infectious disease,^6^ neurology,^4^ and oncology.^2,7^ Studies of the impact of patient engagement in clinical trials have reported that the participation of patients can provide important contributions to study feasibility, rigor, and relevance^19^ and can also have financial value by contributing to the avoidance of protocol amendments and the improvement of enrollment, adherence, and retention.^35^

Patient engagement in RCTs of pain treatments has only recently begun to receive attention, which is unfortunate given the biopsychosocial nature of pain^17^ and the “mosaic” of individual differences that makes pain personal.^14^ Few studies have examined patient preferences regarding analgesic medications,^39,41,60^ and fewer still have evaluated preferences for various characteristics of analgesic clinical trials,^57^ for example, the use of financial incentives and electronic prompts to improve retention.^8^ Such studies of patient preferences can provide an evidence base that can inform patient engagement in clinical trials. Given the valuable contributions that patient engagement can make across all aspects of clinical trials, it will undoubtedly become a standard feature of RCTs of pain treatments in the coming years. Importantly, patient engagement has the potential to improve assay sensitivity by addressing many of the critically important issues discussed elsewhere in the ACTTION Guide, including patient recruitment, training, and adherence; the determination of clinically meaningful differences; and the prevention of missing data.

It is also important to recognize that in addition to patients, there are multiple other important stakeholders whose input and involvement should also be considered throughout the different phases of clinical trials, from design to dissemination. These include but are not limited to family members and caregivers, community-based clinicians, third-party payers, regional health care systems, and government policy makers.

## 3. Discerning hype from substance

Clinical trials of treatments for pain have a long and distinguished history. The earliest clinical trials not only identified analgesic medications and their efficacious dosages but, importantly, also contributed to the development of clinical trial designs and methods that came to be used throughout medicine. The ground-breaking investigators who designed and conducted these early analgesic trials recognized that research methods have a major impact on the ability of a clinical trial to evaluate treatment efficacy and that various sources of bias must be identified and addressed. Over 60 years ago, Lasagna^32,33^ emphasized the crucial roles of randomization and double-blind methods, and Modell and Houde^37^ recognized the importance of multiple research design and participant factors, including “(1) pharmacodynamic actions, (2) dosage, (3) choice of subject, (4) use of controls, (5) collection of data, (6) sensitivity of the method, (7) placebo actions, (8) bias, and (9) forces extraneous to the experiment.”

Presaging the advent of the Delphi polls and evidence-based consensus meetings of the present, Lasagna et al. surveyed active analgesic clinical trial investigators in 1983 about their preferred pain models, experimental designs, subject eligibility criteria, data collection methods, outcome measures, and statistical analyses.^58^ In addition, they examined data from several acute pain trials to compare the ability of different outcome measures and analyses to detect treatment effects. What they concluded over 35 years ago—that agreement on clinical trial methods and analyses “should help to reduce discrepancies between the reported results of different investigators”—remains just as true today.

These analgesic trial pioneers understood the importance of study methods and data analysis, and recognition of the fundamental roles of research design and statistical principles became widespread as experience conducting RCTs grew. Major recent advances in the design, analysis, interpretation, and reporting of clinical trials have involved efforts to at least limit if not completely prevent conclusions that are erroneous, biased, or misleading.^46^ As Fleming^16^ has emphasized, “discerning hype from substance” is necessary because investigators often have a “driving goal to establish benefit,” and the presence of selective reporting of outcomes and analyses in clinical trial publications is, unfortunately, common. The desire to report favorable results can be an important source of bias, and the goal of an RCT should be to “determine whether the experimental intervention is safe and effective” rather than to prove that it is.^16^ One of the most important approaches to mitigating bias is prespecification of the clinical trial's primary outcome and analysis and ensuring that this information is included when the trial is registered on www.clinicaltrials.gov or a similar registry.^16,62^ Exploratory analyses should be clearly described as such and considered hypothesis-generating and requiring confirmation because “if you torture data long enough, they will confess”.^16^

It is beyond the scope of this brief article to discuss the many other important issues involved in improving the rigor and reproducibility of clinical trials of pain treatments; these issues require attention not only by those who conduct clinical research but also by those who seek to apply the results in clinical practice. Specific recommendations for the preparation and review of publications of pain clinical trials can be found in Gewandter et al.,^24^ and many of the other articles in the ACTTION Guide and recent publications^22,34,56^ describe additional approaches to reducing bias in clinical trials of pain treatments and ensuring that the results are as informative as possible.

## 4. Conclusion: the crucial importance of mentoring

In the introduction to part I, we dedicated the ACTTION Guide to Clinical Trials of Pain Treatments to Mitchell Max. We would like to conclude the introduction to part II by emphasizing the crucial importance of mentoring in contributing to the prevention and treatment of pain. Mentoring is not only a source of meaningful advances in research and treatment, but it also ensures continuity. There is no better example of this than Dr. Kathleen Foley, who mentored a generation of distinguished pain investigators, including Mitchell Max, and he is, in turn, considered a very important mentor by one of the ACTTION Guide editors (R.H.D.).

## Disclosures

The Analgesic, Anesthetic, and Addiction Clinical Trial Translations, Innovations, Opportunities, and Networks (ACTTION) public-private partnership with the US Food and Drug Administration (FDA) has provided financial support for the preparation, publication, and distribution of this series of articles. ACTTION has received research grants, contracts, and other support from the FDA, multiple pharmaceutical and device companies, philanthropy, royalties, and other sources (a list of ACTTION's industry sponsors is available at http://www.acttion.org/partners).
